# Characterizing Sleep Differences in Children With and Without Sensory Sensitivities

**DOI:** 10.3389/fpsyg.2022.875766

**Published:** 2022-06-22

**Authors:** Amy G. Hartman, Sarah McKendry, Adriane Soehner, Stefanie Bodison, Murat Akcakaya, Dilhari DeAlmeida, Roxanna Bendixen

**Affiliations:** ^1^Department of Occupational Therapy, University of Pittsburgh, Pittsburgh, PA, United States; ^2^Department of Psychiatry, University of Pittsburgh, Pittsburgh, PA, United States; ^3^Department of Occupational Therapy, University of Florida, Gainesville, FL, United States; ^4^Department of Electrical and Computer Engineering, University of Pittsburgh, Pittsburgh, PA, United States; ^5^Department of Health Information Management, University of Pittsburgh, Pittsburgh, PA, United States

**Keywords:** sensory processing disorder, sleep, children, routine, participation, sensory sensitivity

## Abstract

**Objectives:**

Individuals register and react to daily sensory stimuli differently, which influences participation in occupations. Sleep is a foundational nightly occupation that impacts overall health and development in children. Emerging research suggests that certain sensory processing patterns, specifically sensory sensitivities, may have a negative impact on sleep health in children. In this study, we aimed to (i) characterize sleep in children with and without sensory sensitivities and (ii) examine the relationship between sensory processing patterns (using the Sensory Profile-2) and sleep using validated parent- and child-reported questionnaires. We hypothesized that children with sensory sensitivities will exhibit more difficulties with sleep.

**Methods:**

We recruited 22 children (ages 6–10) with sensory sensitivities (SS) and 33 children without sensory sensitivities (NSS) to complete validated sleep and sensory processing questionnaires: the Children’s Sleep Habits Questionnaire (CSHQ), Sleep Self-Report (SSR), and Sensory Profile-2.

**Results:**

Children with SS had significantly more sleep behaviors reported by both parents (*p* < 0.001, *g* = 1.11) and children (*p* < 0.001, *g* = 1.17) compared to children with NSS. Specifically, children with SS had higher frequencies of sleep anxiety (*p* = 0.004, *g* = 0.79), bedtime resistance (*p* = 0.001, *g* = 0.83), and sleep onset delay (*p* = 0.003, *g* = 0.95). Spearman’s ρ correlations indicated significant positive correlations between parent- and child-reported sleep. Children with SS showed a larger association and greater variability between sleep and sensory processing compared to their peers. Significant positive correlations between parent-reported sleep behaviors and sensory sensitive and avoiding patterns were identified for both children with SS and NSS. Child-reported sleep behaviors were most strongly associated with sensitive and avoiding patterns for children with NSS and seeking patterns for children with SS.

**Conclusion:**

We present evidence that sleep is impacted for children with SS to a greater extent than children with NSS. We also identified that a child’s sensory processing pattern may be an important contributor to sleep problems in children with and without sensory sensitivities. Sleep concerns should be addressed within routine care for children with sensory sensitivities. Future studies will inform specific sleep intervention targets most salient for children with SS and other sensory processing patterns.

## Introduction

Sensory processing, or multisensory integration, occurs in specific areas of the brain as sensory input from the external environment is transformed into usable data, supporting our ability to act in the world (Bundy and Lane, 2019). Epidemiological studies estimate that 5–16% of children in the general population experience sensory processing patterns that impact their daily life (Ayres, 1971; Ahn et al., 2004; Dunn et al., 2016; Galiana-Simal et al., 2020; Mulligan et al., 2021).

Using Dunn’s Sensory Processing Framework (2014), sensory processing patterns can be characterized in four quadrants: (i) low registration, (ii) sensory seeking, (iii) sensory sensitivity, and (iv) sensory avoiding. The low registration and sensory seeking patterns are characterized by their high neurological thresholds for sensory input. Children with higher thresholds often tolerate busy environments more easily than those with low thresholds. They can miss sensory information like verbal cues during school or details in a more complex activity. Children with sensory sensitivity and sensory avoiding patterns have lower neurological thresholds and tend to register and attend to more sensory input than others. They can be very detail-oriented and can flourish with consistent routines that allow them to predict the sensory input they will experience. Each of these sensory processing patterns influence participation in many areas of occupation, such as activities of daily living, play and leisure, and education (Koenig and Rudney, 2010; Cohn et al., 2014; Schaaf et al., 2015; Dunn et al., 2016).

Sleep is an area of occupation that is of interest in healthcare and occupational therapy science. Emerging research suggests that certain sensory processing patterns, specifically sensory sensitivities, may have a negative impact on sleep health for typically developing children (Rajaei et al., 2020), children with attention-deficit hyperactivity disorder (ADHD; Mimouni-Bloch et al., 2021), and children with autism (Tzischinsky et al., 2018). However, the literature has yet to assess sleep in children with predominate sensory sensitivities, which is necessary to begin to disentangle sensory processing difficulties from other neurological differences in special populations and uncover its impact on sleep.

We hypothesize that children with a low neurological threshold, that is, children who are sensory sensitive or sensory avoiders, will exhibit more difficulties with sleep processes. Sleep requires a shift from awake and alert to a relaxed state that allows one to transition to sleep. This process involves complex processes involving biological (Jones, 2020), psychological (Carskadon, 2002), social (Belísio et al., 2010), environmental (Caddick et al., 2018), and family factors (Gregory et al., 2005; Meltzer and Montgomery-Downs, 2011). Children with lower neurological thresholds can experience high sensitivity to sensory information and are more prone to hyperarousal (McIntosh et al., 1999; Lane et al., 2010; Koziol et al., 2011). It is our hypothesis that children who experience these sensory processing patterns find it difficult to calm down to fall asleep at night, or sleep-onset.

We chose to focus on children with predominate tactile (touch) and oral-tactile sensory sensitivities for our study because emerging evidence has identified tactile sensitivity as a potential key contributor to the reported sleep problems in children with autism (Tzischinsky et al., 2018), fetal alcohol spectrum disorder (Wengel et al., 2011), and typically developing children (Shochat et al., 2009).

The goal of this study was twofold. First, we aimed to characterize parent- and child-reported sleep in children (ages 6–10 years old) with reported sensory sensitivities (SS) compared to children without sensory sensitivities (NSS). Using data from validated parent- and child-reported questionnaires, we identified differences found between groups in common bedtime experiences. We specifically investigated reported sleep-onset difficulties for both groups using parent- and child-reported questionnaires and expected to see higher rates of sleep problems reported for children with SS.

Second, we aimed to examine the association between sleep behaviors and sensory processing patterns (sensitivity, avoidance, low registration, seeking) for each group. We specifically hypothesized that there would be a significant association between sleep behaviors and the lower neurological threshold patterns in both groups. Further, we expected to see a greater association between each sensory processing pattern and sleep behaviors for children with SS compared to peers with NSS.

## Materials and Methods

### Study Design

This cross-sectional, observational study utilized validated parent-, and child-reported questionnaires to characterize sleep in children with and without sensory sensitivities. All procedures and consent forms were approved by the University of Pittsburgh’s Institutional Review Board (STUDY20050082).

### Participants

Children between the ages of 6 and 10 years old in the United States and their families were recruited to take part in this remote research study. An *a priori* sample size calculation using the Children’s Sleep Habits Questionnaire (CSHQ) total score indicates a total sample of 17–20 participants in each group would achieve at 95% power to capture important differences between groups. Interested families were screened and consented by the PI (first author) over the phone. Caregivers (all identifying as parents) reported participating in at least 4 nights of their child’s bedtime routine each week. All participating children did not have known sleep disorders and had not engaged in behavioral sleep intervention in the past, or while participating in this research study.

Two groups of children were recruited for this study: children with sensory sensitivities (SS) and children without sensory sensitivities (NSS). Children recruited for the NSS group reported no diagnoses or sensory processing difficulties that impact their daily life. Children recruited for the SS group reported tactile and oral-tactile sensitivities, established by answering “yes” to 6 of the 8 tactile and oral-tactile sensitivity questions posed in the screening process (taken from the Sensory Profile-2 Questionnaire, see Supplementary Appendix A). Children with a diagnosis of autism, ADHD, or Down’s syndrome were excluded from this study as these diagnoses have different components (e.g., neurological, medical) that may impact sleep.

### Protocol

Upon enrollment in the study, all parents and children completed sleep and sensory processing related questionnaires reflecting on the past month: Children’s Sleep Habits Questionnaire (parent-report, CSHQ; Owens et al., 2000a), Child’s Sleep Self-Report (child-report, SSR; Owens et al., 2000b), and the Sensory Profile-2 (parent-report, SP2; Dunn, 2014). Questionnaires and a demographics survey were sent electronically using REDCap software (Clinical and Translational Sciences Institute at the University of Pittsburgh Grant Number UL1-TR-001857). Study data were collected and managed using REDCap, an electronic data capture tool hosted at the University of Pittsburgh (Harris et al., 2009, 2019). REDCap (Research Electronic Data Capture) is a secure, web-based software platform designed to support data capture for research studies. Prior to completing the questionnaires, parents were instructed to allow their child to complete the Sleep Self Report on their own, helping only if their child needs help reading or understanding the questions.

### Outcome Measures

All questionnaire data were reviewed by the study team for completeness. Participants who missed questions were contacted to complete these items.

#### Demographic Questionnaire

A parent-reported demographics survey was developed by the study team to capture important characterizations for each participant. Age, sex, race, ethnicity, and geographic location (e.g., rural, suburban, urban) information was collected for both the parent and child. Parents were asked if their child was currently taking medication and the timing of medication and if any medications or supplements were being taken to aid sleep.

School information was collected, specifically the child’s grade and if school was virtual, hybrid, in-person, homeschool, or another form of schooling. Parents also reported if this year’s school situation was different than what is typical for their child (e.g., before the pandemic) in order to understand if a significant change in schooling could impact sleep.

#### Children’s Sleep Habits Questionnaire

The CSHQ is a parent reported questionnaire that includes 33 unique items reflecting on a child’s sleep over the past month. Questions are scored on a 3-point Likert scale (Rarely, Sometimes, Usually) with higher scores indicating worse sleep. Six items are reverse scored (items 1, 2, 7, 9, 10, 28). The data produce 8 subscale scores: bedtime resistance (6 items), sleep duration (3 items), night waking (3 items), sleep onset delay (1 item), sleep anxiety (4 items), parasomnias (7 items), sleep disordered breathing (3 items), and daytime sleepiness (8 items). Two questions are found in both the bedtime resistance and sleep anxiety subsections, creating a total of 35 questions in the questionnaire. A total score on the CSHQ consists of the sum of 33 unique items. Internal consistency coefficients of the CSHQ are near (0.68) or above (0.78) acceptable standards for the community and clinical samples, respectively (Owens et al., 2000a). A cut-point of 41 correctly identifies 80% of children with clinically significant sleep problems (Owens et al., 2000c).

#### Sleep Self-Report

The Sleep Self-Report (SSR) is a 26-item, 1-week retrospective survey designed to be administered to school aged children between 6 and 12 years (Owens et al., 2000b). This questionnaire is designed to capture domains similar to the CSHQ (parent-report). This tool produces three subscales: bedtime behavior (12 items), sleep behavior (7 items), and daytime sleepiness (4 items). Each item is rated on a 3-point scale (Usually, Sometimes, Never) with a higher score indicating more disturbed sleep. All items are summed for a total score. Internal consistency coefficient is acceptable (0.88) (Owens et al., 2000b).

#### Child Sensory Profile-2

The Child Sensory Profile 2 (SP-2) is a newly updated caregiver-reported questionnaire that evaluates the child’s neurological threshold and self-regulation continuums (Dunn, 2014). The original Sensory Profile has an over 90% discrimination rate between neurodivergent (e.g., children with ASD, ADHD) and neurotypical children (Ermer and Dunn, 1998). The updated SP-2 is found to significantly discriminate between vulnerable populations at a similar rate as the original version (Dunn, 2014). National normative data for clinical and population-based samples are available (Dunn, 2014). The SP-2 uses 86 items scored on a 5-point scale of “Almost Always” (5 points), “Frequently,” “Half the Time,” “Occasionally,” and “Almost never” (1 point). A “Does not apply” option (0 points) is also available in the instances that parents have not observed the behavior in question. Items can be summed to produce *quadrant* subsections (seeking, avoiding, sensitivity, and registration) or *sensory* subsections (auditory, visual, touch, movement, oral, and behavior).

For this study we utilized the *quadrant* scores as measurements of four distinct sensory processing patterns. Within each quadrant, higher scores indicate more frequent sensory behaviors. The sensory sensitivity quadrant characterizes the degree to which a child detects sensory input.

### Statistical Analysis

Data were exported from REDCap and analyzed using Stata/SE (version 17.0; StataCorp 2021). We examined the data for influential outliers and adjusted statistical testing to accommodate for non-influential outliers. No influential outliers were identified.

#### Demographics Analysis

Participants were separated by group (SS and NSS) based on screening questions. Student’s *t*-test or Chi-squared tests were used to compare groups on the demographic variables of age, sex, race, ethnicity, and geographic location to ensure these variables were similar across groups. Additionally, we examined rate of general medication use, medication or supplement use to aid sleep, and frequency of special education services (school based and outpatient) to further characterize our groups.

#### Characterizing Sleep by Group

Means and standard deviations of total scores and subsection or quadrant scores of each questionnaire were calculated and compared by group. To understand the significance of the differences between groups, Student’s *t*-tests or the non-parametric alternatives and Hedges’ *g* effect size estimations for unequal groups were computed. Hedges’ *g* is an effect size that is better suited for our small sample and unequal group sizes. Effect size interpretation for the social sciences when comparing group differences typically indicates *g* > 0.41 as a minimum effect size representing practically significant effect, *g* > 1.15 as a moderate effect, and *g* > 2.70 as a strong effect (Ferguson, 2009). Considering our multiple variables and comparisons, a probability level of *p* < 0.01 was set *a priori* to indicate significance.

#### Examining Relationship Between Sensory Processing Patterns and Sleep Problems

For the second aim of this study, we examined the relationship between each sensory processing pattern and reported sleep behaviors for each group (SS and NSS). Sensory processing pattern scores were calculated using the SP-2 scoring criteria and correlated with the sleep questionnaires total scores. All variables were correlated using Spearman’s ρ with the probability level of *p* < 0.05 set *a priori* to indicate significance. We used Mukaka’s guidance of correlation coefficient magnitude in medical research of 0.70–1.00 indicating high correlations, 0.50–0.70 indicating moderate correlations, 0.30–0.50 indicating low correlations, and < 0.30 indicating negligible correlations (Mukaka, 2012). Scatter plots were used to visualize the data for each group and a fitted line was drawn to represent the magnitude and direction of the relationship between each sensory processing pattern (quadrant) and sleep total score. Due to our small sample size, we were unable to quantify the amount of variance in reported sleep each sensory quadrant explained in each group, however, we can visualize the data to inform future larger studies.

## Results

### Demographics

A total of 57 parents and children were consented for this study, 23 in the SS group and 34 in the NSS group. Prior to the completion of the study, one participant asked to be withdrawn due to a family move and one participant was lost in follow-up, resulting in 22 participants in the SS group and 33 in the NSS group. All participants were recruited between September 2021 and December 2021 when families were transitioning into the school year during the COVID-19 pandemic. Children were reported to have had at least 2 weeks of school prior to starting our study with hopes that they have at least begun to adjust to the new schedule.

The groups were similar in child age, parent age, and child sex, but differed on racial and ethnic diversity (see Table 1). The SS group also had more diversity in location (urban, suburban, and rural). Additionally, the majority of both groups were living in the mid-west or eastern areas of the United States; however, the SS group did have one participant in the south and two participants in the west.

**TABLE 1:** Demographics.

	SS group (*n* = 22)	NSS group (*n* = 33)	*p*-value
Parent age (*SD*)	38.36 (3.57)	37.33 (4.59)	0.487
Child age (*SD*)	7.46 (1.44)	7.46 (1.65)	0.999
Child sex^†^			0.375
Male (%)	14 (64%)	17 (52%)	
Female (%)	8 (36%)	16 (48%)	
**Child race and ethnicity**			
Black/African American, Hispanic	2 (9%)	0 (0%)	0.078
Black/African American, Non-Hispanic	4 (18%)	0 (0%)	0.011*
White/Caucasian, Hispanic	1 (5%)	0 (0%)	0.216
White/Caucasian, Non-Hispanic	12 (55%)	30 (91%)	0.002**
Other/multiple	4 (18%)	3 (9%)	0.322
**Geographic location**			
Urban	9 (41%)	3 (9%)	0.005*
Suburban	9 (41%)	24 (73%)	0.018*
Rural	4 (18%)	6 (18%)	0.999
Child use of melatonin for sleep (%)^†^	7 (32%)	3 (9%)	0.032*
**Special education services** ^†^			
Children receiving special education services (%)	10 (45%)	5 (15%)	0.013*
Children receiving outpatient therapy services (%)	8 (36%)	2 (6%)	0.004**

*Student’s t-test performed unless otherwise indicated.*

^†^ *Chi Squared test performed.*

*p < 0.05, **p < 0.01.

Parents of children with SS reported diagnoses of anxiety (13%), trauma (13%), behavior-related diagnoses (e.g., Oppositional Defiance Disorder; 8.6%), and developmental delay (4.3%). While none of the children in this study had a diagnosis of ADHD or Autism, two children with SS were reported to take Strattera or Ritalin daily (common ADHD medications). Additionally, two children were reported to take Prozac and Zoloft (common antidepression medication) however, no children were reported to have a diagnosis of depression. Within the group of children with NSS, one child was reported to have a diagnosis of anxiety (3%) and one was reported to have asthma (3%). The only medications reported to be taken for children with NSS were allergy medication, multivitamins, magnesium and Vitamin D supplements, and melatonin. All other children with NSS did not report a diagnosis that impacts their daily life.

Another key difference between the groups was the number of children using medications or supplements to support sleep. Melatonin was used significantly more often by children with SS (*n* = 7/22, 32%) when compared to children with NSS (*n* = 3/33, 9%, χ*^2^* = 4.58, *p* = 0.032). Parents who reported use of melatonin used small doses (ranging from 0.5 to 2.5 mg) between 30 and 60 min prior to bed.

While we did not directly ask about adoption during our study, 5 children with SS were reported to have been adopted. No children with NSS were said to have been adopted, however, parents were not directly asked. Additionally, more children with SS received special education services at school (SS = 45%, NSS = 15%, χ*^2^* = 6.11, *p* = 0.013) and outside of school (SS = 36%, NSS = 6%, χ*^2^* = 8.15, *p* = 0.004). These services included occupational therapy, physical therapy, speech therapy, and therapies from psychologists and psychiatrists. Parents did not specify the goal areas of these services; however, it was indicated that all children had not received interventions related to sleep in the past or currently.

### Characterizing Sleep

#### Child’s Perception of Sleep

A total score of the SSR was also compared between groups to characterize a child’s perception of their sleep (Table 2). These data met all assumptions for parametric testing and therefore were compared using Student’s *t*-test. Children with SS scored significantly higher (i.e., indication of increased difficulties) in overall sleep scoring compared to children with NSS [*Mean*_SS_ = 42.18, *SD*_SS_ = 8.26, *Mean*_NSS_ = 33.55, *SD*_NSS_ = 6.71, *t*(53) = −4.26, *p* < 0.001, *g* = 1.17]. When examining subsection scores, we found a statistically significant difference between the bedtime [*t*(53) = −3.68, *p* < 0.001, *g* = 1.01] and sleep behavior (*U* = −3.63, *p* < 0.001, *g* = 1.23) subscales. In addition to the questions included in the total score, children were asked “*do you have trouble sleeping?”* as part of the SSR. For children with SS, 64% (14/22) indicated that they had trouble sleeping. For children with NSS, only 21% (7/33) indicated that they had difficulty sleeping (χ^2^ = 6.49, *p* = 0.011).

**TABLE 2:** Comparison of groups.

	SS group (*n* = 22)	NSS group (*n* = 33)	*p*-value	Hedges’ *g*
Sleep self-report total score^†^	42.18 (8.26)	33.55 (6.71)	<0.001**	1.17^‡‡^
Bedtime^†^	22.45 (4.78)	12.97 (4.19)	<0.001**	1.01^‡^
Sleep behavior	12.68 (3.05)	9.64 (2.01)	<0.001**	1.23^‡‡^
Daytime sleepiness^†^	7.05 (1.84)	5.94 (1.58)	0.021	0.66^‡^
Children’s sleep habits Questionnaire total score	54.91 (10.00)	45.12 (7.27)	<0.001**	1.11^‡^
Bedtime resistance	9.64 (2.66)	7.61 (2.32)	0.001*	0.83^‡^
Sleep onset delay	2.09 (0.81)	1.45 (0.56)	0.003*	0.95^‡^
Sleep duration	5.05 (2.01)	4.12 (1.39)	0.095	0.56^‡^
Sleep anxiety	7.41 (2.40)	5.64 (2.12)	0.004*	0.79^‡^
Night waking	4.82 (1.87)	3.70 (0.92)	0.018	0.81^‡^
Parasomnias	10.68 (2.42)	8.42 (1.52)	<0.001**	1.17^‡‡^
Sleep disordered breathing	3.59 (1.01)	3.39 (0.70)	0.493	0.24
Daytime sleepiness	11.64 (3.90)	10.79 (2.70)	0.557	0.26
**Sensory profile 2**				
Seeking^†^	55.41 (20.99)	31.36 (11.61)	<0.001**	1.50^‡‡^
Avoiding	57.00 (18.04)	32.24 (11.47)	<0.001**	1.72^‡‡^
Sensitivity	50.14 (16.00)	27.76 (9.15)	<0.001**	1.81^‡‡^
Registration^†^	49.45 (16.09)	30.88 (10.16)	<0.001**	1.45^‡‡^

*Means presented with standard deviations in parentheses with higher scores indicating higher frequencies of problem behavior. Non-parametric Mann-Whitney U-test used until indicated. Hedges’ g corrected for uneven groups was used to calculate effect size.*

^†^ *Student’s t-test was used.*

^‡^ *Effect size interpretation for social sciences typically is as follows (Ferguson, 2009).*

^‡^ *g > 0.41 minimum effect, ^‡‡^g > 1.15 moderate effect.*

*p < 0.01, **p < 0.001.

#### Parent’s Perception of Child’s Sleep

To characterize a parent’s perception of their child’s sleep, we calculated and compared the total score of the CSHQ by group. Due to the non-normality of these data, we used non-parametric Mann-Whitney *U*-tests to compare groups (Table 2). Parents reported a statistically significant difference in overall sleep, with children with SS (*Mean*_SS_ = 54.91, *SD*_SS_ = 10.00) scoring higher (i.e., indication of increased difficulties) than children with NSS (*Mean*_NSS_ = 45.12, *SD*_NSS_ = 7.27; *U* = −3.41, *p* = 0.001, *g* = 1.11). Further analysis of subsections indicated that parents of children with SS identified higher frequencies of bedtime resistance, sleep onset delay, sleep anxiety, night awakenings, and parasomnias. Both groups scored similarly on sleep duration, sleep disordered breathing, and daytime sleepiness subsections. Ninety-one percent of total scores for children with SS exceeded the cut-point of 41, indicating clinically significant sleep problems (Owens et al., 2000a). Interestingly, 67% of total scores for children with NSS exceeded the cut-point, a higher rate than what is reported in the literature (Markovich et al., 2015).

#### Correlations Between Parent and Child Reported Sleep

In a *post hoc* analysis, we correlated parent and child reported sleep total scores by group to see the relationship between both perspective of the child’s sleep. Interestingly, we found that children with SS and their parents had very low, non-significant correlations between their reported of sleep (ρ = 0.14, *p* = 0.526). Children with NSS and their parents showed a small and significant positive correlation between their total scores (ρ = 0.40, *p* = 0.020).

### Relationship Between Sleep and Sensory Processing Patterns

#### Characterizing Sensory Processing Patterns in Each Group

Children with SS and NSS differed significantly on the four quadrant scores of the SP-2, with children with SS scoring significantly higher for each quadrant score (Table 2). Twenty (91%) children with SS scored higher than at least 1 standard deviation (or “More than others” on the tool) from the mean on one of the quadrants, and 11 (50%) scored 2 standard deviations (or “Much more than others”) from the mean on at least one quadrant.

On average, children with NSS scored within one standard deviation from the mean in all sensory quadrants. However, 8 children (24%) with NSS had at least one quadrant score falling one standard deviation above the mean, and 2 participants (6%) scored higher than 2 standard deviations above the mean on a quadrant score.

#### Correlations Between Sleep and Sensory Processing Patterns

**FIGURE 1:**
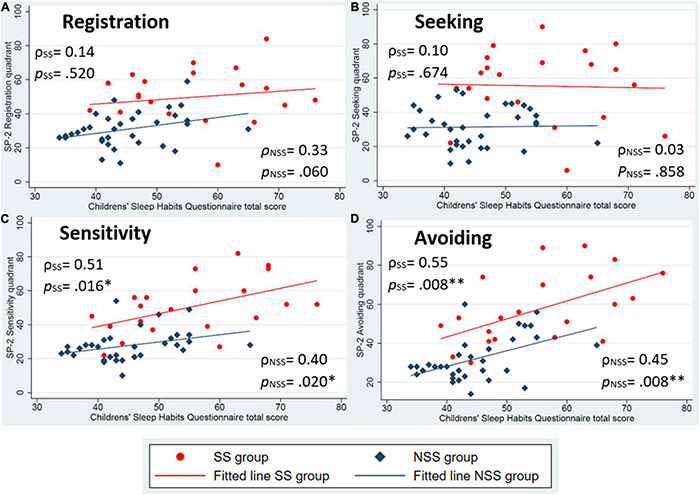
Scatter plots of parent-reported sleep (Children’s Sleep Habits Questionnaire) and sensory processing patterns (Sensory Profile-2 quadrant scores). **(A)** Is the registration pattern, **(B)** is the seeking pattern, **(C)** is the sensitivity pattern, and **(D)** is the avoiding pattern. Spearman’s ρ correlations and *p*-values presented for each group. *p < 0.05, ^**^p < 0.01.

**FIGURE 2:**
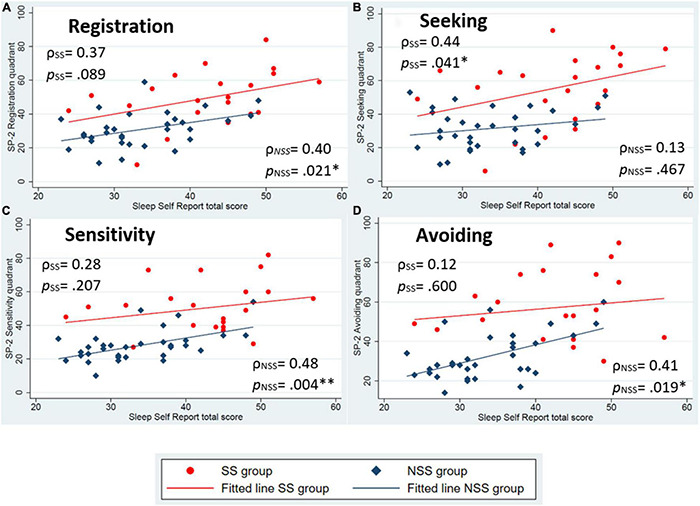
Scatter plots of child-reported sleep (Sleep Self Report) and sensory processing patterns (Sensory Profile-2 quadrant scores). **(A)** Is the registration pattern, **(B)** is the seeking pattern, **(C)** is the sensitivity pattern, and **(D)** is the avoiding pattern. Spearman’s ρ correlations and *p*-values presented for each group. *p < 0.05, ^**^p < 0.01.

We then examined the correlations between sleep and sensory variables by group. Two compilations of scatter plots are presented in Figures 1, 2. Figure 1 represents the correlation of parent-reported sleep by each sensory quadrant score by group and Figure 2 presents the correlations between child-reported sleep and each sensory quadrant score. From these scatter plots and fitted lines, potential differences in the magnitude and significance of the relationship between reported sleep problems and sensory processing patterns can be explored.

Aligning with our hypothesis, both groups had statistically significant associations between parent-reported sleep and low neurological threshold patterns (Figures 1C,D). For child-reported sleep, a significant association was found in low neurological threshold patterns for only children with NSS (Figures 2C,D). Contrary to our hypothesis, children with SS did not identify statistically significant associations with low neurological threshold patterns. Rather they identified sensory seeking as a significantly associated pattern related to sleep (Figure 2B).

Additionally, we compared the magnitude of the associations within each sensory processing pattern by group. Using parent-reported sleep behaviors, larger correlations were found for children with SS compared to children with NSS for all sensory patterns except the low registration pattern. This aligns with our hypothesis and suggests that sensory processing is slightly more strongly associated with sleep behaviors in children with SS compared to peers. Child-reported sleep, however, was found to be more strongly associated with high neurological threshold patterns for children with SS (Figures 2A,B) and low neurological threshold patterns for children with NSS (Figures 2C,D).

Additionally, a larger variability can also be seen in children with SS compared to children with NSS, as evident by the larger spread of data in this group. We see a shift toward higher scores on both of the sleep variables and each of the sensory processing pattern scores for children with SS in these plots, indicating that children with SS more frequently endorse the sensory and sleep behaviors noted in these questionnaires.

## Discussion

First, this preliminary cross-sectional, observational study adds to the science by characterizing sleep behaviors in a novel group: children with sensory sensitivities who do not have a diagnosis of autism or attention-deficit hyperactivity disorder. We compare these data to children without sensory sensitivities (NSS) to understand the areas in which these groups differ. We found significantly increased sleep difficulties (e.g., more frequent bedtime resistance, parasomnias, sleep anxiety) for children with SS compared to children with NSS. The current results align with previous findings in neurodiverse children; studies have found higher prevalence of sleep problems in children with ADHD (Langberg et al., 2020; Mimouni-Bloch et al., 2021) and autism (Souders et al., 2017; Manelis-Baram et al., 2021). Both of these groups have high rates of co-occurring sensory processing patterns that impact daily life (Ghanizadeh, 2011; Ausderau et al., 2016; Kadwa et al., 2019; Dellapiazza et al., 2021).

Second, we noted that parents reported a small to moderate and significant correlation between the lower neurological threshold patterns in both groups. Parents of children with SS reported slightly larger correlations than parents of children with NSS, aligning with our hypothesis that sensory processing patterns are more strongly associated with sleep difficulties for children with SS. When examining child-reported sleep correlations, we found that only children with NSS report similar small, significant, and positive correlations between sleep behaviors and low neurological threshold patterns. Children with SS, on the other hand, report negligible correlations between sleep behaviors and low neurological threshold patterns and small-moderate correlations between sleep behaviors and the higher neurological threshold patterns, a finding that opposed our hypothesis.

As other sleep research has found, we see interesting discrepancies between parent and child reported sleep (Owens et al., 2000a; Short et al., 2013). As research continues to explore sleep in pediatric populations, it is critical to incorporate both parent and child reported perspectives of sleep to construct a more complete picture of the components of sleep. Using these two methods, we can see that while both parents and children identify higher rates of sleep difficulties for children with SS compared to children with NSS, parent and child reports correlate with different sensory processing patterns.

### Relationship Between Sleep and Sensory Processing

Characterizing the relationship between sleep and sensory processing patterns has been of recent interest in the pediatric sleep research community. Rajaei et al. (2020) recently published a large, cross-sectional study with typically developing children in Tehran correlating the Persian version of the CSHQ with Sensory Profile quadrant scores. They found small but highly significant correlations between CSHQ total scores and all sensory process patterns.

Recent studies have also identified similar findings in other diagnostic populations. Manelis-Baram et al. (2021) highlight similar significant correlations between parent-reported sleep (CSHQ) and sensory processing patterns in young children with autism prior to the pandemic. More frequent endorsement of sensory processing behaviors has also been correlated with more frequent parent-reported sleep problems for children with ADHD (Mimouni-Bloch et al., 2021).

While research continues to home in on specific relationships between sensory processing patterns and sleep in children, our findings of higher reported sleep difficulties for children with SS compared to peers suggests that a sensory sensitive pattern could be a key component to understanding the high rates of sleep problems in children (Hollway and Aman, 2011; Reynolds et al., 2011; Mazurek and Petroski, 2015; Deliens and Peigneux, 2019).

### The Impact on the Occupation of Sleep

Sleep is a critical occupation that requires a skilled transition from wakefulness to sleep (American Occupational Therapy Association, 2020). Our results indicate that children with sensory sensitivities struggle with independence in this transition. More specifically, there is emerging evidence that children with low neurological threshold patterns, or those with sensory sensitivities and sensory avoidance, show the largest relationship with poor sleep outcomes (Shochat et al., 2009; Thomas et al., 2015; Rajaei et al., 2020). Being that sleep is foundational to a child’s health, growth, and development, sleep should routinely be part of care for children with sensory sensitivities.

### Strengths, Limitations, and Future Directions

An important strength of this study was the comparison of children with SS to a peer group with NSS. This allows us to account for some of the current historical (e.g., COVID) and temporal (e.g., time of year) factors that otherwise might influence our outcomes of interest. We also used validated questionnaires that are widely used to characterize sleep and sensory processing patterns, a strength to consider when applying our findings with the larger body of literature.

Our study does have limitations that are important to consider. Our study uses a small sample size that lacked racial and ethnic diversity to mirror the United States’ demographic make-up. A larger, more diverse sample in future research will further uncover the relationships between sleep and sensory processing patterns. Our groups also were significantly different in their geographic location and urbanicity. These variables can lead to differences in exposure to a myriad of environmental sensory stimuli like nighttime light and environmental noise that could negatively influence sleep in children who are more attune to sensory stimuli.

Our groups also differed in their use of medications or supplements to support sleep. We found a significantly higher rate of melatonin use for children with SS compared with children with NSS. This could be reflective of the significantly higher rates of sleep behaviors noted for children with SS. Parents and children who identify higher rates of sleep behaviors may turn to medication and supplements more readily to address these problems. It is interesting that despite the higher rates of melatonin use, children with SS still report higher sleep behaviors. Future research could examine melatonin use and its perceived effects for children with SS compared to children with NSS.

It should be noted that four children with SS were taking medications that may impact their sleep (Strattera, Ritalin, Prozac, and Zoloft). There were also a similar number of children in both groups taking allergy medication which may impact sleep for these children. Future research should document more information regarding medication timing and effects parents and children note regarding their daily medication.

Additionally, in this study we purposefully sampled children who were reported to have sensory sensitivities. While this allows for a strong sample of children with lower neurological threshold, children with predominately high neurological thresholds may have been excluded, biasing our findings. Future research should include children with a variety of sensory processing patterns to better understand correlations with sleep.

An important consideration with this study is the timeline of data collection, which took place between September 2021 and December 2021, during the COVID-19 pandemic (Delta variant predominance) in the United States. All but one of our participants were attending school in person at the time of data collection, however, some reported having to recently quarantine at home due to COVID exposure or infection. While the effects of COVID on child sleep is still being explored, some studies conducted at the beginning of the pandemic show an increase in overall sleep duration and sleep quality during the early pandemic (Sharma et al., 2021). However, in our data, we show high rates of sleep problems reported in both children with SS and NSS at this time, which may be a result of the higher level of stress and schedule variabilities due to COVID exposure or illness and quarantine restrictions.

Children’s sleep habits do not occur in a vacuum, but in a dynamic family context; this context is critical for pediatric sleep researchers to remember (Dahl and El-Sheikh, 2007; Meltzer and Montgomery-Downs, 2011). Children with poor sleep often impact the family functioning, just as family functioning can impact a child’s sleep (Meltzer and Montgomery-Downs, 2011). In this study we did not measure family functioning, parental stress, or overall feelings of burden related to child sleep problems. Future research may consider the family dynamic and parental stress and the impact of poor child sleep on family functioning.

This is one of the very few studies characterizing sleep in children with SS who do not have a diagnosis of autism or ADHD, and lays the groundwork for future studies characterizing sleep using objective sleep measures like actigraphy or polysomnography for children with SS. These measurement tools can provide additional information that can support development of targeted sleep intervention for children with each sensory processing pattern. Additionally, exploration of circadian rhythm timing for children with each sensory processing pattern would be an interesting aspect to consider for future research.

## Conclusion

Good sleep is critical for childhood development and overall health. We have found evidence that children with sensory sensitivities experience higher rates of sleep difficulties that can be captured by parent- and child-reported questionnaires. Further, we show positive correlations between parent-reported sleep behaviors and low neurological threshold patterns (e.g., sensitivities and avoiding) in both groups. These data indicate children who are reported to have more frequent sensory-related behaviors endorse more frequent bedtime problems. We believe our study provides a step toward uncovering specific sleep intervention targets and will contribute to improvement in everyday care for children with sensory sensitivities.

## Data Availability Statement

The raw data supporting the conclusions of this article will be made available by the authors, without undue reservation.

## Ethics Statement

The studies involving human participants were reviewed and approved by the University of Pittsburgh’s Institutional Review Board. Written informed consent to participate in this study was provided by the participants’ legal guardian/next of kin.

## Author Contributions

AH developed the original study concept, completed the data analysis, and wrote the first draft of the manuscript. RB, AS, SB, MA, and DD contributed to the study development and analysis plan. AH and SM completed the data collection. All authors approved the final manuscript.

## Conflict of Interest

The authors declare that the research was conducted in the absence of any commercial or financial relationships that could be construed as a potential conflict of interest.

## Publisher’s Note

All claims expressed in this article are solely those of the authors and do not necessarily represent those of their affiliated organizations, or those of the publisher, the editors and the reviewers. Any product that may be evaluated in this article, or claim that may be made by its manufacturer, is not guaranteed or endorsed by the publisher.
