# Supplementary material for: Characterizing Sleep Differences in Children With and Without Sensory Sensitivities
Source: Front Psychol. 2022 Jun 22;13:875766. doi: 10.3389/fpsyg.2022.875766 (PMC9257069; doi:10.3389/fpsyg.2022.875766)
Supplement: Supplementary file 1 [file Data_Sheet_1.docx]

# Appendix A: Screening questions

Sensory Over-responsivity screening questions taken from the Sensory Profile-2. A parent must answer ‘yes’ to at least 6 of the following questions in order to be included in the SS group:

1. Does your child show distress during grooming (for example, fights or cries during haircutting, face washing, fingernail cutting)?
2. Is your child particular about the types of clothing s/he will wear?
3. Does your child show an emotional or aggressive response to being touched?
4. Does your child become anxious when standing close to others (for example when in a line?)
5. Does your child rub or scratch a part of the body that has been touched?
6. Does your child gag easily from certain food textures or food utensils in his/her mouth?
7. Does your child limit his/herself to certain food textures?
